# The Maternal Nutrition and Offspring's Epigenome (MANOE) study: a prospective, monocentric, observational study.

**DOI:** 10.1186/2049-3258-73-S1-P41

**Published:** 2015-09-17

**Authors:** Sara Pauwels, Inge Huybrechts, Roland Devlieger, Gudrun Koppen, Lode Godderis

**Affiliations:** 1 KU Leuven, Leuven, Belgium; 2 International Agency for Research on Cancer (IARC), Lyon, France; 3 KU Leuven/ UZ Leuven, Leuven, Belgium; 4 Flemish Institute of Technological Research (VITO), Mol, Belgium; 5 KU Leuven/IDEWE, Leuven, Belgium

## Introduction

Epigenetic modifications have the ability to change the susceptibility to metabolic diseases like obesity. DNA methylation can change during a life course due to environmental exposures like diet.

## Aim

To determine the effects of dietary methyl-group intake during pregnancy on the DNA methylation pattern of mother and child. In addition, the association between the DNA methylation pattern of the mother and child on body composition/weight gain of the infant during the first year will be studied.

## Methods

We have recruited 166 and 95 expectant mothers and fathers respectively, who are followed up in UZ Leuven. *Validated questionnaires* are used to obtain information about lifestyle and environmental factors that can influence DNA methylation. A *food-frequency questionnaire* was developed and validated to categorize women in groups according to their methyl-group intake. Women fill out a *7-day dietary record* to have information about macro- and micronutrient intake. Body composition is followed up by means of the *bio-electrical impedance* method. *Blood samples* are collected during pregnancy and until 1 year postpartum. *Cord blood* is taken and *mouth epithelial cells* are obtained from the infants at 6 and 12 months. Samples will be analyzed for global DNA (de)methylation by liquid chromatography-mass spectrometry and specific target genes involved in DNA (de)methylation and genes linked with obesity/adiposity by pyrosequencing.

## Results

Our first results give an indication that the intake of methyl-groups during pregnancy is stable, except for a decrease in total folate intake in the second and third trimester. Also, global DNA methylation analysis shows that maternal methylation is rather stable over pregnancy, with a small decrease (-0.17%,p=0.03) in methylation by the end of pregnancy.

## Discussion

With this study we will gain insight on the effect of maternal nutrition on offspring DNA methylation and potentially identify DNA methylation biomarkers at birth that can mediate problems with metabolism/obesity.

